# Genome-Wide Analysis of Circular RNAs Mediated ceRNA Regulation in Porcine Embryonic Muscle Development

**DOI:** 10.3389/fcell.2019.00289

**Published:** 2019-11-19

**Authors:** Linjun Hong, Ting Gu, Yanjuan He, Chen Zhou, Qun Hu, Xingwang Wang, Enqin Zheng, Sixiu Huang, Zheng Xu, Jie Yang, Huaqiang Yang, Zicong Li, Dewu Liu, Gengyuan Cai, Zhenfang Wu

**Affiliations:** ^1^National Engineering Research Center for Breeding Swine Industry, College of Animal Science, South China Agricultural University, Guangzhou, China; ^2^Guangdong Provincial Key Laboratory of Agro-Animal Genomics and Molecular Breeding, College of Animal Science, South China Agricultural University, Guangzhou, China

**Keywords:** porcine, circRNAs, ceRNA, embryo, skeletal muscle

## Abstract

Many circular RNAs (circRNAs) have been discovered in various tissues and cell types in pig. However, the temporal expression pattern of circRNAs during porcine embryonic muscle development remains unclear. Here, we present a panorama view of circRNA expression in embryonic muscle development at 33-, 65-, and 90-days post-coitus (dpc) from Duroc pigs. An unbiased analysis reveals that more than 5,000 circRNAs specifically express in embryonic muscle development. The amount and complexity of circRNA expression is most pronounced in skeletal muscle at day 33 of gestation. Our circRNAs annotation analyses show that “hot-spot” genes produce multiple circRNA isoforms and RNA binding protein (RBPs) may regulate the biogenesis of circRNAs. Furthermore, we observed that host genes of differentially expressed circRNA across porcine muscle development are enriched in skeletal muscle function. A competing endogenous RNA (ceRNA) network analysis of circRNAs reveals that circRNAs regulate muscle gene expression by functioning as miRNA sponges. Finally, our experimental validation demonstrated that circTUT7 regulate the expression of HMG20B in a ceRNA mechanism. Our analyses show that circRNAs are dynamically expressed and interacting with muscle genes through ceRNA manner, suggesting their critical functions in embryonic skeletal muscle development.

## Introduction

Pork is one of the most important source of meat in the world, which accounts for nearly 40% of all meat consumed and closely related to human health (Wang et al., 2015; Sun et al., 2018). As living standards improve, lean meats are favorite amongst people following low-fat diets and low calorie (Sun et al., 2018). Skeletal muscle is the major component of lean meat. The study of the mechanism underlying skeletal muscle development is beneficial to genetic improvement for lean meat quality and quantity. Therefore, the most critical goal of meat production science is analyzing and understanding of the development of porcine skeletal muscle. Additionally, it is also beneficial to understanding human muscular atrophy and muscle regeneration because of genomic characteristics, physiological, and anatomical likenesses between human and pig (Wernersson et al., 2005; Verma et al., 2011).

Skeletal muscle is post-mitotic tissue composited of multinucleated skeletal myofibers, which differentiated from mononucleated muscle cells (Yin et al., 2013). The embryonic and postnatal development of skeletal muscle affects meat production and growth rate in pigs (Tang et al., 2007; Zhao et al., 2015). Previous studies showed that the emergence of primary and secondary fibers at distinct embryonic stages is essential during the skeletal muscle development of pigs (Wigmore and Stickland, 1983). At the same time, the total number of fibers are constant in the postnatal stage, and postnatal muscle growth is through hypertrophy of myofibers, or conversion of myofiber types (Ashmore et al., 1973; Suzuki and Cassens, 1980). Recent studies showed that transcriptomic analysis based on poly A + RNA-seq provide a comprehensive view of the expression dynamics of mRNAs during muscle development (Tang et al., 2007; Zhao et al., 2015). Besides, numerous studies demonstrated that non-coding RNAs is expressed explicitly across different tissues and play critical roles during different development stages of porcine. However, the standard poly A + RNA-seq technology only catches the tip of the iceberg of the entire spectrum of non-coding RNA, a large set of non-coding RNA warranting further investigation.

Circular RNAs (circRNAs) as a large class of non-coding RNA, which are generated from precursor mRNA back-splicing (Jeck et al., 2013; Zhang et al., 2014; Starke et al., 2015; Wang and Wang, 2015; Li X. et al., 2018). With the advance of ribosome depleted and RNase R + RNA-Seq, thousands of circRNAs in mammalian cells have been discovered (Jeck et al., 2013; Jeck and Sharpless, 2014). Although the majority of circRNAs still lack functional annotation, recent studies reported that circRNAs might play essential roles in gene regulation (Li X. et al., 2018). For instance, a few abundant circRNAs such as ciRS-7, which is preferentially expressed in human and mouse brains (Hansen et al., 2013; Memczak et al., 2013), could act as miRNA sponges. A set of intron-containing circRNAs was shown to regulate RNA polymerase II (Pol II) transcription (Li et al., 2015). Besides, circRNAs may play important roles in different biological processes (Li X. et al., 2018). Thousands of circRNAs are expressed at high levels in the brain (Rybak-Wolf et al., 2015; You et al., 2015). Many such circRNAs are up-regulated during neurogenesis (Rybak-Wolf et al., 2015) and are enriched in synaptogenesis than their linear counterparts (You et al., 2015). However, the functions of circRNAs upon mammalian embryonic development remains mostly unknown, requiring further investigation.

Recently, there were several researches on the effect of circRNAs on muscle development. For instance, Liang et al. (2017) identified 149 circRNAs potentially associated with postnatal muscle growth using ribosome depleted RNA-seq; Morten T. et al. demonstrated that circRNAs are highly abundant and dynamically expressed in a spatiotemporal manner in fetal brain of porcine using time series total RNA-seq datasets (Venø et al., 2015). However, total (rRNA-depleted) RNA-seq restrains the discovery of a completed catalog of circRNAs and the molecular functions of circRNAs in embryonic muscle development remain mostly unknown, warranting further exploration.

In this study, we performed RNase R + RNA-seq in three distinct stages of embryonic skeletal muscle development (33, 65, and 90 days prenatal) from Duroc pigs (one of the world-class lean meat type pigs), which have been widely used as the terminal male parent of the DLY (Duroc × Landrace × Yorkshire) commercial pigs due to its excellent lean ratio on growth traits. With the advance of RNase R + RNA-seq, we first identified circRNAs embryonic skeletal muscle and found that many circRNAs specifically expressed in different embryonic stages. The motif enrichment analysis of flanking regions showed that many RNA binding proteins (RBPs) tend to regulate circRNAs biogenesis. Further circRNAs expression analysis across developmental stages indicated the potential functional role of circRNAs in modulating muscle development. Besides, the competing endogenous RNA (ceRNA) interaction network analysis demonstrated that circRNAs might regulate critical regulators of muscle development. In summary, our study provides a comprehensive temporal map of circRNA expression in embryonic skeletal muscle development of porcine, which is beneficial for studying the regulatory mechanisms and expanding the molecular genetics of muscle fiber development.

## Materials and Methods

### Animals and Muscle Tissue Preparation

Duroc gilts were obtained from the breeding pig farm of Guangdong Wen’s Foodstuffs Group Co., Ltd. (Yunfu, China). Nine gilts were checked for estrus twice daily and were artificially inseminated at the onset of estrus (Day 0) and again 12 h later. The gilts were slaughtered at a local slaughterhouse on days 33, 65, and 90 of gestation (*n* = 3 gilts/day of gestation). The longissimus muscle tissue was rapidly dissected from each fetus (three samples per time-point). Nine muscle samples were taken and prepared. All samples were immediately snap frozen in liquid nitrogen and stored at −80°C until further use.

### Library Preparation and RNA Sequencing

Total RNA was extracted from the nine frozen longissimus muscle tissues using TRIzol reagent (Invitrogen, CA, United States) according to the manufacturer’s instructions. For poly A + RNA-seq, Oligo (dT) selection was performed twice by using Dynal magnetic beads (Invitrogen) according to the manufacturer’s protocol, then sequencing by NovaSeq. For linear RNA depleted RNA-seq, RNase + R treatment was carried out as described previously (Zhang et al., 2013). Briefly, purified RNAs were incubated with 40 U of RNase R (Epicenter) for 3 h at 37°C and then were subjected to purification with TRIzol. The RNA integrity and concentration were assessed using the Agilent 2100 Bioanalyzer (Agilent Technologies, Palo Alto, CA, United States) and met the experimental requirement of the illumina sequencing platform. The quality of all the sample solutions had RNA integrity numbers (RIN) 7.0 and 28S/18S 1.0. RNA libraries were constructed and sequencing was carried out by HiSeq X ten. The raw reads produced in this study were deposited in the NCBI Sequence Read Archive (SRA), the records can be accessed by accession numbers PRJNA556496 and PRJNA556325.

### RNA-seq Analysis

RNA-seq raw reads were subjected to adapter trimming and quality filtering (Phred score >20) using TrimGalore (Martin, 2011), then filtered reads were mapped to the porcine genome (Sscrofa11.1) using STAR v2.6 with default parameters (Dobin et al., 2013). Reads mapped to multiple locations within the genome, only one alignment record was retained. The expression level of each gene was quantified by fragment per kilobase exon model per million sequencing reads (FPKM) using Cufflinks (Trapnell et al., 2012). Genome and annotation files were downloaded from Ensembl database^1^ (Aken et al., 2016). RNA-seq read coverage was visualized for select genes in the Integrative Genomics Viewer (Thorvaldsdóttir et al., 2013) (Supplementary Tables S1–S3).

### Motif Enrichment Analysis

The flanking regions of back splicing site with circRNAs were retrieved from the porcine genome, then the short, ungapped motifs relatively enriched in these regions compared with shuffled sequences were detected using dreme (Bailey, 2011). To associate the enriched motifs to potential RBPs, all enriched motifs were compared against a database of known motifs using Tomtom (Gupta et al., 2007; Bailey et al., 2009). The top 20 target motifs with the most significant matches to the query motif were identified as the potential RBPs, which might regulate the biogenesis of circRNAs. The logo plots of enriched motifs were generated by weblogo in MEME suite (Bailey et al., 2009; Ray et al., 2013).

### Identification of ceRNA Pairs

To identify ceRNA pairs among circRNAs and protein-coding genes (PCGs), we first screen potential miRNA target binding sites at 3’ UTR of PCGs and circRNAs using Miranda (Betel et al., 2010)with default parameters. Then, we adopted a previous method to examine whether ceRNA pairs are significantly co-regulated by miRNAs. Briefly, we determine ceRNA pairs by hypergeometric test, which is comprised of four parameters: (i) *N* is the number of miRNAs used to infer target genes; (ii) *K* is the number of miRNAs that interact with the chosen gene of interest; (iii) *n* is the number of miRNAs that interact with the candidate ceRNA of the chosen gene; and (iv) *c* is the number of miRNA bound these two genes at the same time. The test produces a *P*-value for each potential ceRNA pair using the following formula:

$$P=\sum_{i=c}^{\text{min}\,\left(K,n\right)}\frac{\left(\begin{array}{c} K\\ i \end{array}\right)\,\left(\begin{array}{c} N-K\\ n-i \end{array}\right)}{\left(\begin{array}{c} N\\ n \end{array}\right)}$$

In hypergeometric test, multiple miRNAs from the same family were merged into one and every miRNA family were counted only once, even though it has multiple binding sites at the same 3’UTR of PCGs or circRNAs. All *P*-values were subjected to false discovery rate (FDR) correction with Benjamini/Hochberg procedure (Noble, 2009). We determined a PCG and a circRNA as ceRNA pair if the *P*-value is less than 0.0001.

### Network Analysis

All ceRNA pairs can be represented as a graph with PCGs and circRNAs as nodes, the ceRNA pairs as edges. To construct ceRNA regulatory network, we first filtered PCGs with expression level less than 1 (FPKM) and circRNAs supported with <3 back splicing reads. Then, we retrieved sub-network involving myogenesis for further analysis. The myogenesis related genes were downloaded from MSigDB (Liberzon et al., 2011). The network graph was generated by Cytoscape (Smoot et al., 2011).

### Heatmaps

The expression values of circRNAs were log2 transformed if they express in at least one stages of embryonic muscle development. Then, the heatmaps were generated by heatmap package (Kolde, 2019). The absent expression values were replaced with the lowest score.

### Quantitative RT-PCR

RNA extraction, cDNA synthesis and quantitative PCR were performed as previously described (Li G. et al., 2018, p. 70; Ao et al., 2019). For each time-point quantitative PCR was done on three biological replicates, each with three technical replicates. PCR primers spanning back-splice junction were used to validate the selected circRNAs. The sequence specificity of the primer sequences was evaluated by BLAST and the detailed information was included in Supplementary Table S6.

### Dual-Luciferase Reporter Assay

The sequences of circRNA 10:29037750| 29038934, which is located on chromosome 10 and derived from terminal uridylyl transferase 7 (TUT7), and thus we named it circTUT7, and HMG20B-3’UTR and their corresponding mutant versions without ssc-miR-30a-3p binding sites were synthesized and subcloned into psiCHECK-2.0 dual luciferase reporter plasmid (Promega, Madison, WI, United States), termed circTUT7-WT, circTUT7-Mut, HMG20B 3’UTR-WT, and HMG20B 3’UTR-Mut, respectively. All these plasmids were validated by sequencing. The dual-luciferase assays were conducted as previously described (Hong et al., 2019). Briefly, porcine kidney cell lines (PK15) were cultured in DMEM plus 15% fetal bovine serum (FBS) and then plated into a 96-well plate. The miR-30a-3p mimics, mimics negative control (NC) or miR-30a-3p inhibitors were co-transfected into cells with 3’-UTR dual-luciferase vector using Lipofectamine 3000 (Invitrogen). Cells were collected 24 h after transfection, the relative luciferase activity was examined by Dual Luciferase Assay Kit (Promega, Madison, WI, United States) in accordance with the manufacturer’s protocols. Three replicates were performed for each transfection.

### Vector Construction and Transfection

The circRNA overexpression vector was constructed using the linear sequence of circTUT7 amplified from porcine muscle by PCR. They were then cloned into pCDciR2.1 vector (Supplementary Figure S2, Geneseed Biotech, China) in accordance with the manufacturer’s protocol using the *Kpn*I and *Bam*HI restriction sites. Porcine fetal fibroblast (PFFs) were collected from 30-day-old Duroc fetuses and cultured in dishes with DMEM plus 15% FBS at 38.5°C as previously described (Yang et al., 2018). miRNA mimics, mimics NC and circRNA overexpression vector were transiently transfected into PFFs using Lipofectamine 3000 reagent (Invitrogen, United States). PFFs were collected 48 h after transfection, and proteins were extracted for further experiments.

### Western Blot Analysis

Protein extraction and western blot were conducted as previously described (Hong et al., 2019). Briefly, total proteins from PFFs were homogenized using RIPA buffer. Protein concentrations were determined using the BCA Protein Assay Kit (Thermo Pierce, United States). Proteins were separated by 10% sodium dodecyl sulfate polyacrylamide gel electrophoresis, transferred to a polyvinylidene fluoride membrane (Millipore, United States), and then incubated with antibodies (HMG20B, Abcam; GAPDH, Abcam) overnight at 4°C and then with HRP-conjugated secondary antibody for 1 h at room temperature. Pictures were captured by an imaging system (UVP, United States) and quantification analysis was performed by ImageJ 1.45 software (NIH Image).

### Statistical Analysis

Data from the results of q-PCR, Dual Luciferase Reporter Assays and Western Blotting were analyzed using SPSS software version 21.0 (SPSS, Inc., Chicago, IL, United States). Paired *t*-tests and two-way analyses of variance were performed to analyze the relative expression of genes and the luciferase activity. A *P*-value < 0.05 was considered a significant difference.

## Results

### Identification of Circular RNAs During Embryonic Skeletal Muscle Development

**FIGURE 1:**
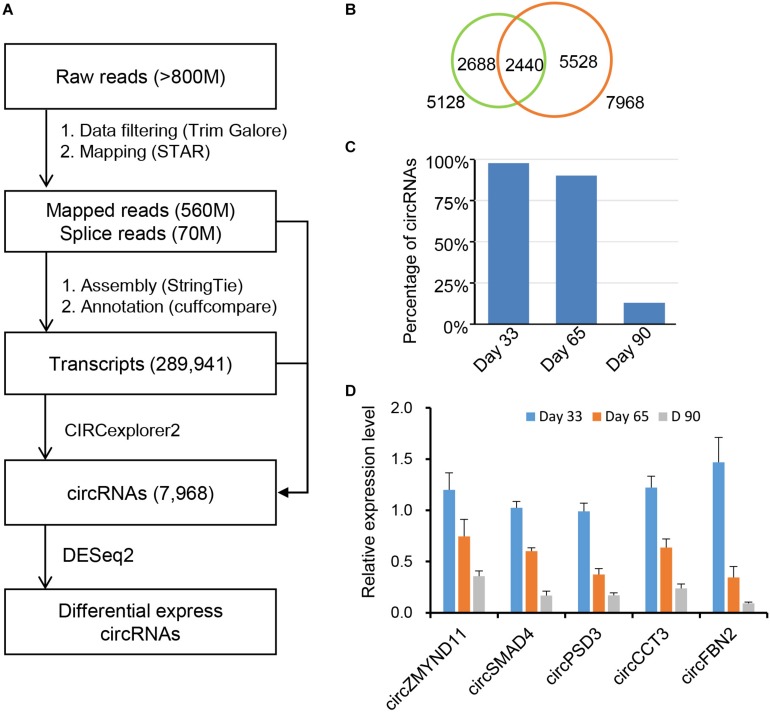
Identification of circRNAs in skeletal muscles at embryonic development. **(A)** A pipeline for circRNA identification. **(B)** Overlapping of the newly assembled circRNAs and the reported circRNAs. **(C)** The distribution of circRNAs on day 33, day 65, and day 90 of gestation. **(D)** RT-qPCR validation of circRNAs during embryonic muscle development, results are presented as mean ± SD (three independent replicates per group).

Recent studies have demonstrated that circRNAs play an essential role in regulating postnatal skeletal muscle development (Venø et al., 2015; Liang et al., 2017), but their roles in embryonic skeletal muscle development remain mostly unknown, warranting further exploration. To address this question, we applied RNase R + RNA-seq, which can deplete linear RNA and enrich circRNAs; thus, it can obtain a comprehensive view of circRNAs during different time points of embryonic muscle development in porcine. We applied a data processing pipeline to identify circRNAs. Briefly, the low-quality bases and adapter sequence of the raw reads were filtered using TrimGalore with default parameters (Martin, 2011). Then, all filtered reads were mapped back to the porcine reference genome, ∼560 million reads can unique mapped and >70 million of them are spanning the splicing regions (Figure 1A and Supplementary Table S1). To *ab initio* identify transcripts in the porcine genome, we applied StringTie (Pertea et al., 2015) to assemble the transcriptome, and resulted in 289,941 transcripts. Using the transcriptome as the reference, we adopted CIRCexporer2 to predict circRNAs transcription across the genome (Zhang X.-O. et al., 2016); we finally got 84,693 circRNAs and we quantify all circRNAs across 3-time courses. To generate a confident set of circRNAs, we only retained circRNA with FPKM >0.05 (Venø et al., 2015); these steps result in 7,968 circRNAs (Figure 1A). We further examine the overlapping between the newly assembled circRNAs and the known circRNAs in porcine circRNAs databases. As a result, we found that 5,528 circRNAs is expressed explicitly in our dataset, suggesting RNase R + RNA-seq can provide a comprehensive view of circRNAs during embryonic muscle development (Figure 1B). Furthermore, we found that a high percentage of circRNAs is expressed on day 33 of gestations, indicating that their crucial functions in the initiation stage of skeletal muscle development (Figure 1C). Further RT-qPCR experiments showed that the relative expression of circRNAs dynamic express during embryonic development, suggesting the functionalities of identified circRNAs (Figure 1D and Table 1). Collectively, our comprehensive analysis indicated that circRNAs play an essential role during time courses of embryonic muscle development.

**TABLE 1:** Validation of RNA-seq results by using quantitative RT-PCR.

**circRNA ID**	**Symbol**	**Methods**	**Day 33**	**Day 65**	**Day 90**	**Correlation**
10:69201376-69235186	circZMYND11	q-PCR	1.20	0.75	0.36	0.97
		FPKM	8.90	7.44	4.43	
1:100589850-100613174	circSMAD4	q-PCR	1.03	0.60	0.17	0.99
		FPKM	6.74	3.53	1.70	
17:13008092-13031589	cirD3	q-PCR	0.99	0.37	0.17	0.94
		FPKM	8.77	6.90	4.58	
4:93738637-93747422	circCCT3	q-PCR	1.22	0.64	0.24	0.96
		FPKM	8.80	7.23	4.08	
2:131354294-131363905	circFBN2	q-PCR	1.47	0.35	0.09	0.85
		FPKM	7.08	5.45	1.99	

### Characterization of Circular RNAs

**FIGURE 2:**
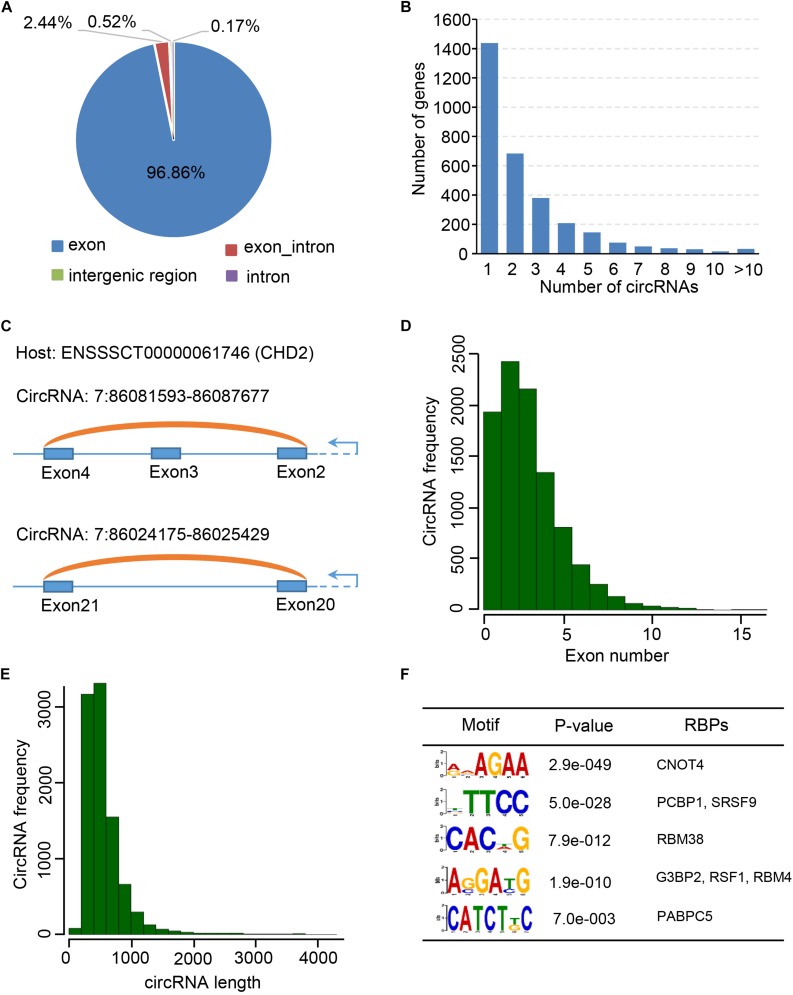
Characterization of circular RNAs. **(A)** The pie chart shows the genic distribution of circRNAs. **(B)** The bar chart shows the correlation between gene number and the number of circRNAs. **(C)** CircRNA: 7:86081593-86087677 and circRNA: 7:86024175-86025429 transcribed from CHD2 gene. **(D)** Bar chart shows the exon number distribution of circRNAs. **(E)** Bar chart shows the length distribution of circRNAs. **(F)** RBP binding motif enriched in the flanking region of circRNAs.

Previous studies showed that many circRNAs contain multiple exons and derives from exon regions (Jeck et al., 2013; Zhang et al., 2014). However, sequence characteristics of circRNAs expressed during embryonic skeletal muscle development remain unclear. We firstly examined the distribution of circRNAs using Ensembl gene annotation as the reference annotation. As a result, we observed that circRNAs are extensively transcribed from exon regions (96.86%), whereas a small fraction (<3%) is originated from exon intron splicing junction, intergenic and intron regions (Figure 2A), suggesting that circRNAs have potential to regulate the expression of their host genes through ceRNA manner (Zhang et al., 2014; Fan et al., 2015; Venø et al., 2015). Expectedly, more than 1,400 host genes contain only one circRNA, while a small proportion can generate more than five circRNAs, which indicates that the circRNAs may specifically regulate the function of their host gene in post-transcriptional level. Moreover, our genome wide analysis showed that the expression level of 2,782 (38.7%) of 7,190 circRNA-mRNA pairs are correlated (| pearson *r*| > 0.9) during embryonic muscle development (Supplementary Table S8). When we explored the expression of circRNA and their host genes in different time points, no significant correlation was detected among them in a specific time point (Supplementary Figure S1). Besides, we indeed found that two circRNAs (7:86081593-86087677 and 7:86024175-86025429) were generated from CHD2 gene (Figures 2B,C), which is a key regulator alter gene expression by modification of chromatin structure (Harada et al., 2012; Zhang K. et al., 2016). To explore molecular characteristics of circRNAs, we then explore the length and exon number distribution of circRNAs. In consistent with previous studies (Liang et al., 2017), a large majority of circRNAs is with no more than five exons, with a median exon number of three (Figure 2D). In the meantime, a similar distribution of circRNA exonic sequence lengths was observed, with a median length of 509 nucleotides, and most porcine circRNAs are shorter than 2 kb (Figure 2E). Even though many circRNAs have been discovered in different cell type and tissues, the biogenesis mechanism of circRNAs is mostly unknown. RBPs play important roles in regulating the functions of RNAs. We suspected that RBPs enriched in the flanking region of circRNAs junction site may participate in the biogenesis of circRNAs in different biological systems. Interestingly, we found that the binding motifs of CNOT4, PCBP1, SRSF9, RBM38, G3BP2, RSF1, RBM4, and PABPC5 are enriched in flanking region of circRNAs junction sites and express during embryonic muscle development, indicating that their associated RPBs may play functional roles in circRNA biogenesis (Figure 2F and Supplementary Table S7). In summary, our result provided a comprehensive characterization of circRNAs during embryonic muscle development.

### Expression Dynamics of Circular RNAs in Embryonic Skeletal Muscle Development

**FIGURE 3:**
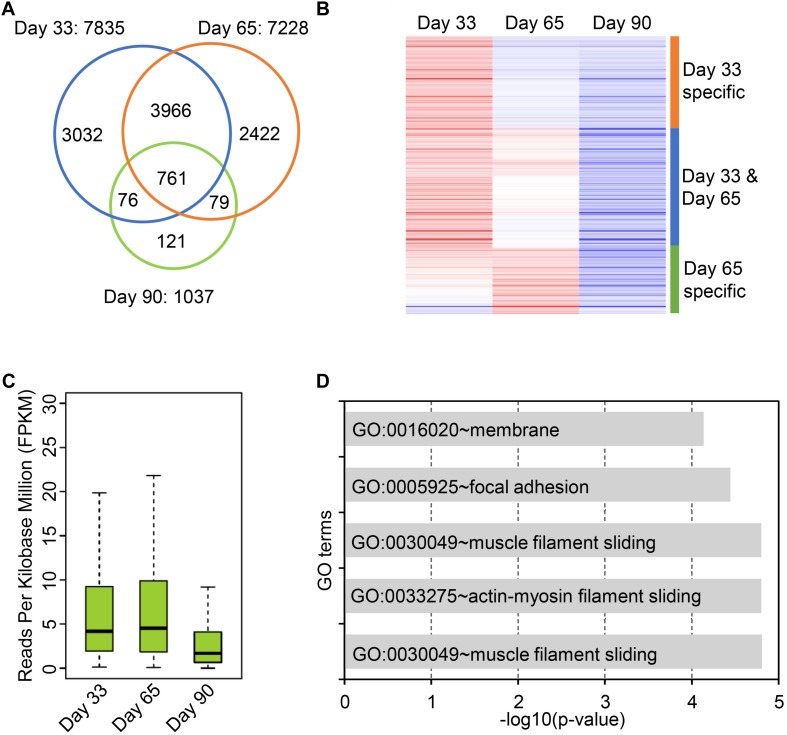
Expression dynamics of circular RNAs in embryonic skeletal muscle development. **(A)** Venn diagram shows the overlap of circRNAs on 33, 65, and 90 days of gestation. **(B)** Heatmap shows the expression dynamics of circRNAs on 33, 65, and 90 days of gestation. **(C)** The boxplot shows the expression of circRNAs on 33, 65, and 90 days of gestation. **(D)** Bar chart shows GO term enrichment analysis of the host gene of circRNAs.

Circular RNAs are differentially expressed during postnatal skeletal muscle development (Liang et al., 2017), but little study focuses on their temporal expression during embryonic skeletal muscle development. To this end, we sought to explore expression profiles of circRNAs in muscle development at 33, 65, and 90 days of gestation. As a result, 7,968 skeletal muscle circRNAs were obtained from three stages; of these circRNAs, 7,835 circRNAs were detected on day 33, whereas 7,228 were detected on day 65 and 1,037 were detected on day 90. 81.5% of circRNAs were detected at only one stage (Figure 3A). To explore the expression dynamics, we carried out the hierarchical clustering of circRNAs expression across three stages. As a result, all circRNAs were clustered into three groups: (1) circRNAs specifically expressed on Day 33; (2) circRNAs expressed on both day 33 and day 65; (3) circRNAs specifically expressed in day 65. A vast majority of circRNAs are with lower expression level on day 90 compared with day 33 and day 65, while a small set of circRNAs (449 of 7,968) are activated in day 90 (Figures 3B,C and Supplementary Table S3). For instance, the expression of circPRIMA1 is elevated in day 65, circKDM5B is continuous up-regulated during embryonic muscle development and the expression of circCOL14A1 remains constant (Supplementary Figures S3A–C). Recent studies showed that circRNAs might regulate their host gene expression (Venø et al., 2015; Barrett and Salzman, 2016; Liang et al., 2017), we thus perform GO enrichment analysis for the host gene of circRNAs to exploring the functions of the host gene of circRNAs. GO enrichment analysis showed that host genes of circRNAs were enriched in GO:0030049 muscle filament sliding and GO:0030239 myofibril assembly, suggesting that circRNAs temporally regulate embryonic muscle development (Figure 3D and Supplementary Table S4). Next, we focused on the host genes of abundant circRNAs in skeletal muscle. In particular, we indeed found that several circRNAs derived from PCGs with crucial roles in skeletal muscle development and muscle function (e.g., MYH3, MYH8, TMOD1, TNNT1, MYBPC1, and MYL6). MYH3 and MYH8 belong to the MYH myosin superfamily. Myosin, the primary component of thick filaments, is widely expressed in mammalian skeletal muscle and involved in muscle contraction, phagocytosis, cell motility, and vesicle transport (Supplementary Table S4). In summary, our analyses demonstrate that circRNAs dynamically express across three stages of muscle development and potential to regulate muscle development by altering the expression of their host genes.

### Exploration of Circular RNA Mediated ceRNA Regulatory Network

**FIGURE 4:**
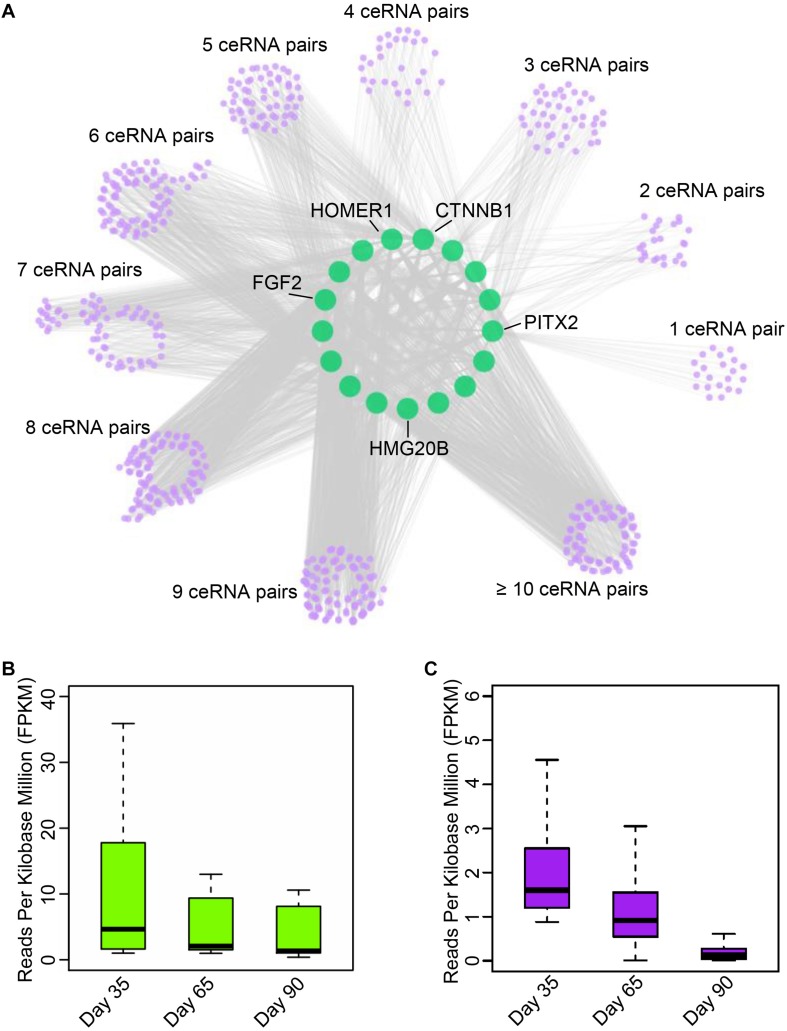
Exploration of circular RNA mediated ceRNA regulatory network. **(A)** Illustration of the ceRNA interaction network. The purple node represents circRNAs; green node represents key genes related to myogenesis. **(B)** The boxplot shows the expression dynamics of key myogenesis genes on 33, 65, and 90 days of gestation. **(C)** The boxplot shows the expression of circRNAs involved in myogenesis on 33, 65, and 90 days of gestation.

Numerous studies have demonstrated that circRNAs function as miRNA sponges to indirectly regulate mRNA abundance (Hansen et al., 2013; Panda, 2018). To establish ceRNA pairs among circRNAs and mRNAs, we performed poly A + RNA-seq and RNase R + RNA-seq in day 33, 65, and 90 of gestation (Supplementary Table S1) and filtered genes with FPKM <1. Furthermore, we predicted miRNA binding sites of circRNAs and 3’UTR of mRNAs using Miranda (Betel et al., 2010). As a result, 7,968 circRNAs were predicted bound by 348 miRNAs, while 10,121 mRNAs were bound by 348 miRNAs. Finally, we adopted a method in previous report (Liang et al., 2017) to detect ceRNA pairs using hypergeometric test. This procedure resulted in a ceRNA network contains 13,142,864 interactions between 2,247 circRNAs and 7,866 mRNAs. Subsequently, we examined the interactions between myogenic genes and circRNAs by incorporating gene interaction network retrieved from STRING database (Szklarczyk et al., 2017). As a result, we found that PITX2, CTNNB1, fibroblast growth factor 2 (FGF2), HOMER1, and HMG20B, which are key regulators during myogenesis, are strongly regulated by multiple circRNAs (Figure 4A and Supplementary Table S5). Particularly, we found circRNA regulates PITX2 expression, which has emerged as a key element involved in the fine-tuning mechanism that regulates skeletal-muscle development as well as the differentiation (Hernandez-Torres et al., 2017). Further expression analysis demonstrated that the myogenic genes are down-regulated during embryonic muscle development, while their corresponding circRNAs are with similar expression patterns (Figures 4B,C). These results suggest that circRNAs could regulate gene expression by functioning as miRNA sponges. Although these *in silico* results should be further investigated *in vivo*, these results illuminate the manner in which circRNAs regulate mRNA abundance through ceRNA mechanism.

### Experimental Validation of circRNA-mRNA-miRNA Regulatory Circuitry

**FIGURE 5:**
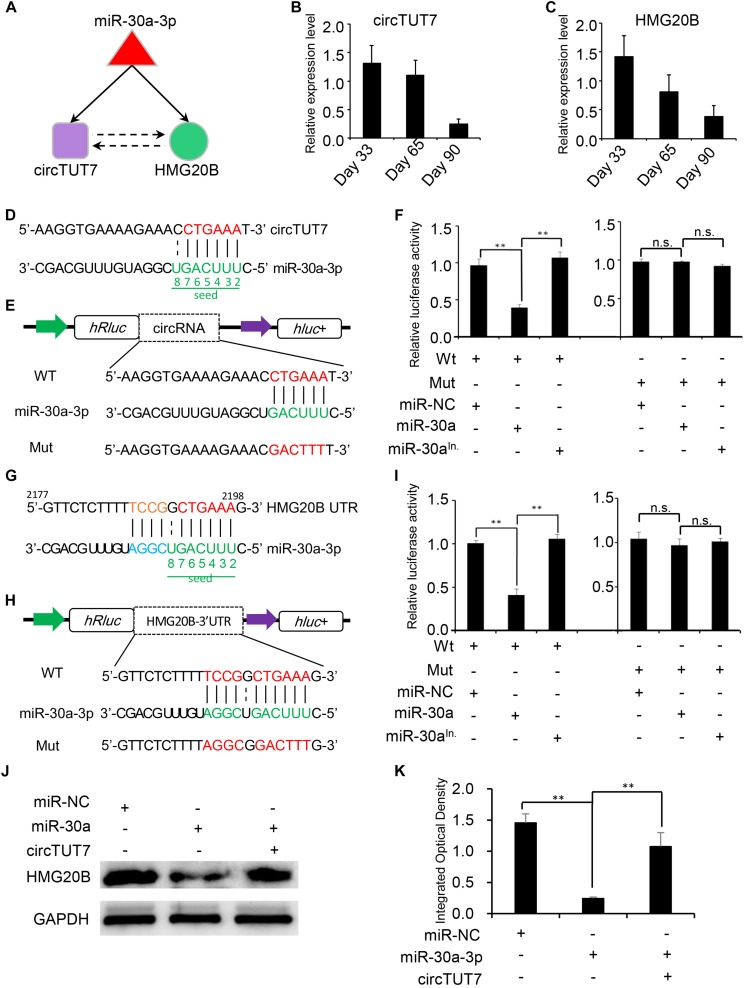
Experimental validation of circRNA-mRNA-miRNA regulatory circuitry. **(A)** Illustration of a three nodes circuitry including miRNA-30a-3p, circTUT7, and HMG20B. The purple node represents circRNAs; green node represents key genes related to myogenesis; the red node represents miRNA. RT-qPCR shows the relative expression level of circTUT7 **(B)** and HMG20B **(C)** during embryonic muscle development. **(D)** Predicted miR-30a-3p binding site on circTUT7. **(E)** The design of luciferase reporter. WT, the WT sequence of circTUT7 contains miR-30a-3p binding site; Mut, the sequence of circTUT7 with mutation in miR-30a-3p binding site. **(F)** PK15 cells were co-transfected with wild-type (WT) or mutant (MUT) luciferase reporters of circTUT7 with miR-30a-3p mimics, mimics negative control (NC) or miRNA inhibitors. The relative levels of firefly luminescence normalized to Renilla luminescence are plotted. Error bars represent SD (*n* = 3). **(G)** Predicted miR-30a-3p binding site on HMG20B-3’UTR. **(H)** The design of luciferase reporter. WT, the WT sequence of HMG20B-3’UTR contains miR-30a-3p binding site; Mut, the sequence of HMG20B-3’UTR with mutation in miR-30a-3p binding site. **(I)** PK15 cells were co-transfected with wild-type (WT) or mutant (MUT) luciferase reporters of HMG20B with miR-30a-3p mimics, mimics NC or miRNA inhibitors. The relative levels of firefly luminescence normalized to Renilla luminescence are plotted. Error bars represent SD (*n* = 3). **(J)** Expression of HMG20B protein in PFFs infected with miRNA mimics, mimics NC and circRNA overexpression vector. **(K)** Results of western blotting were quantified by ImageJ (v1.45). The relative levels of HMG20B protein are plotted, error bars represent SD (*n* = 3). ^∗∗^*P* < 0.01.

Recent studies have demonstrated that a set of sub-graphs contained three nodes are responsible for carrying out specific biological functions (Milo et al., 2002; Alon, 2007; Zhou et al., 2017). Through circRNA network analysis, a regulatory circuitry containing circTUT7, miR-30a-3p and HMG20B was found in our analysis; HMG20B is a component of a BHC histone deacetylase complex involved in histone modification in development (Hakimi et al., 2002). We hypothesized that circTUT7 function as miRNA (miR-30a-3p) sponges to regulate the abundance of HMG20B indirectly (Figure 5A). To validate the regulatory circuitry, we then checked the expression of circTUT7 and HMG20B using RT-qPCR during embryonic muscle development. As a result, the expression of circTUT7 and HMG20B is positively correlated, indicating that circTUT7 may regulate the expression of HMG20B (Figures 5B,C). To further confirm the binding evens among miR-30a-3p, circTUT7, and HMG20B, we screened the potential miR-30a-3p binding site within the sequence of circTUT7 and 3’UTR of HMG20B (Figures 5D–F). To this end, we examined the interaction between miR-30a-3p and circTUT7 using psiCHECK-2.0 dual luciferase reporter assay with integration of circTUT7 overexpression and miR-30a mimic (see section “Materials and Methods”). As expected, we found that miR-30a-3p can significantly decrease the luciferase activity of circTUT7; we also found that miR-30a-3p can significantly decrease the luciferase activity of HMG20B (Figures 5G–I) compared with NCs (miR-NC and miR-30a-3p inhibitor). Furthermore, western blot analysis demonstrated that ectopic expression of miR-30a-3p inhibited the expression HMG20B in PFFs, but the inhibitory effect of miR-30a-3p on HMG20B protein expression could be reversed by over-expression of circTUT7 (Figures 5J,K). These results evidenced that circTUT7 may function as a sponge of miR-30a-3p to regulate the expression of HMG20B. In summary, our results showed that three nodes circRNA mediated regulatory circuitry might play an essential role during skeletal muscle embryonic development.

## Discussion

Recent studies have explored the characteristics of circRNAs across different tissues in pig (Zhao et al., 2015), and the expression dynamic of circRNAs have been investigated during postnatal muscle development (Liang et al., 2017). Those studies are helpful to explore and elucidate the functional roles of circRNAs in muscle development and growth at a genome-wide scale. Moreover, the domestic pig is an excellent model system for the study of embryonic development in mammals (Verma et al., 2011). Thus, circRNAs profiling in our study and others may provide a reference for studies on human muscle tissue development and dysfunction.

Muscle fiber development in pig occurs in two waves, around 33 and 65 days post-coitus (dpc), which involve the formation of primary and secondary fibers (Wigmore and Stickland, 1983; Davoli et al., 2011). The muscle growth is predominantly determined during prenatal skeletal muscle development (Dahmane Gosnak et al., 2010). Even though the previous study showed that circRNAs potentially played an important role in postnatal muscle development, the expression dynamics of circRNAs during embryonic muscle development is unknown (Venø et al., 2015; Liang et al., 2017). In this study, we investigated circRNA expression in primary (33 dpc), secondary (65 dpc) fibers developmental stages and later (90 dpc) developmental stages using RNA sequencing with linear RNA depletion. There are more circRNAs expressed in primary and secondary muscle fibers development compared with later stage, suggesting that circRNAs may involve in the regulation of the proliferation and differentiation of myogenic precursor cells at the earlier stage. Furthermore, RBPs binding motif enrichment analysis showed that RBPs might extensively involve in the biogenesis of circRNAs. Besides, GO enrichment analysis of host genes of circRNAs indicated that the potential functional role of circRNAs in regulating the expression of their host genes. Expression dynamics analysis showed that circRNAs are down-regulated during embryonic muscle development. Collectively, we provided the first panoramic view of circRNA during embryonic muscle development.

Competing endogenous RNA network analysis of myogenic genes showed that circRNA might modulate muscle development. As a result, we found that PITX2, FGF2, CTNNB1, HOMER1, HMG20B were the key regulators during myogenesis that regulated by bundles of circRNAs. Furthermore, we found that PITX2 was regulated by multiple miRNAs, including microRNA-21 and miR-137. Our finding is consistent with previous studies that PITX2 was regulated by microRNA-21 in pituitary tumor, regulated by miR-137 in myogenic differentiation of endometrial mesenchymal stem cells and regulated by miR-644a in esophageal squamous cell carcinoma (Chen H.-S. et al., 2017; Cui et al., 2017, p. 21; Zhang et al., 2017). Additionally, we found FGF2 was regulated by miR-124 and miR-205, suggesting that it may be functional in the muscle and satellite cell development (Ghadiali et al., 2017). Previous study revealed that FGF2 was targeted by circRNA WDR77, then inhibit the cell proliferation and migration in the vascular smooth muscle cells by sponging miR-124 (Chen J. et al., 2017). In summary, our analysis demonstrated that ceRNA regulation plays a crucial role in regulating embryonic development.

**FIGURE 6:**
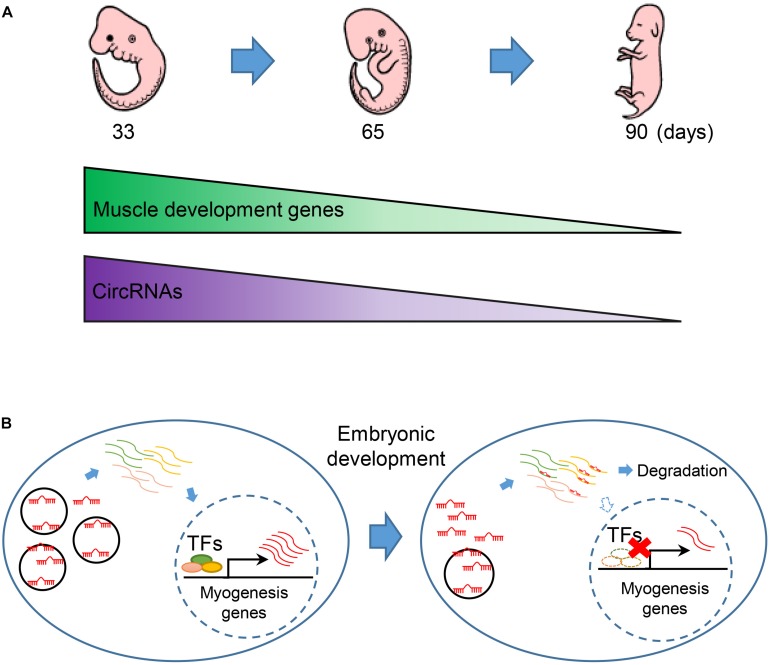
The regulatory model of circRNA during embryonic muscle development. **(A)** The expression of muscle development genes and circRNAs are synergistically express in the three key time points during prenatal skeletal muscle development. **(B)** CircRNAs regulate embryonic development in miRNA sponge manner. Down-regulation of circRNAs thus causes the release of miRNA that regulated the abundance of key transcription factors (TFs) and eventually promotes the expression of muscle genes during embryonic muscle development.

By leveraging advantages of linear RNA depleted, we get a completed map with 7,968 circRNAs, which is larger than current circRNA databases in pig (Liang et al., 2017). Most of the circRNAs are exclusive expression during embryonic development in pig. We observed that circRNAs are continuous down-regulated in embryonic muscle development (Figure 6A). Notably, we found that the expression of muscle development genes is synergistic with circRNAs. Moreover, miRNAs are extensively involved in interactome between circRNAs and muscle development genes. Furthermore, our experimental validation demonstrated that circTUT7 function as miRNA (miR-30a) sponges to regulate the abundance of HMG20B. Thus, we proposed that circRNAs can regulate the abundance of key transcription factors through a ceRNA manner, then fine-tune the expression of muscle-related genes during embryonic muscle development in pig (Figure 6B). Even though we found that circTUT7 could regulate the abundance of HMG20B by ceRNA manner in PFFs, we emphasize that future studies should be done in the porcine primary myoblasts to investigate the biological functions of circRNAs during embryonic muscle development.

## Conclusion

In conclusion, our study provides a comprehensive analysis of ceRNA profiles, which is helpful for further research on human culture, organic evolution, biomedical research, and animal breeding. Therefore, we believe that the circRNA profile and relevance NGS datasets of *Sus scrofa* in this study will benefit the mechanistic investigation of circRNA in mammal.

## Data Availability Statement

The datasets used and analyzed during the current study are available from the corresponding authors on reasonable request. The raw reads produced in this study were deposited in the NCBI Sequence Read Archive (SRA), the records can be accessed by accession numbers PRJNA556496 and PRJNA556325.

## Ethics Statement

The experimental animal procedures were followed in accordance with the approved protocols of South China Agricultural University, Guangdong Province, China for the Biological Studies Animal Care and Use Committee.

## Author Contributions

LH, TG, GC, and ZW conceived and designed the research. YH, CZ, QH, ZX, EZ, SH, JY, and HY collected the samples and performed the experiments. LH, TG, XW, ZL, and DL performed the sequencing analysis and drafted the manuscript. All authors read and approved the final version of the manuscript.

## Conflict of Interest

The authors declare that the research was conducted in the absence of any commercial or financial relationships that could be construed as a potential conflict of interest.

## Footnotes

1 ftp://ftp.ensembl.org/pub/release-75
