# Supplementary figures and images for: Genome-Wide Analysis of Circular RNAs Mediated ceRNA Regulation in Porcine Embryonic Muscle Development
Source: Front Cell Dev Biol. 2019 Nov 19;7:289. doi: 10.3389/fcell.2019.00289 (PMC6877547; doi:10.3389/fcell.2019.00289)

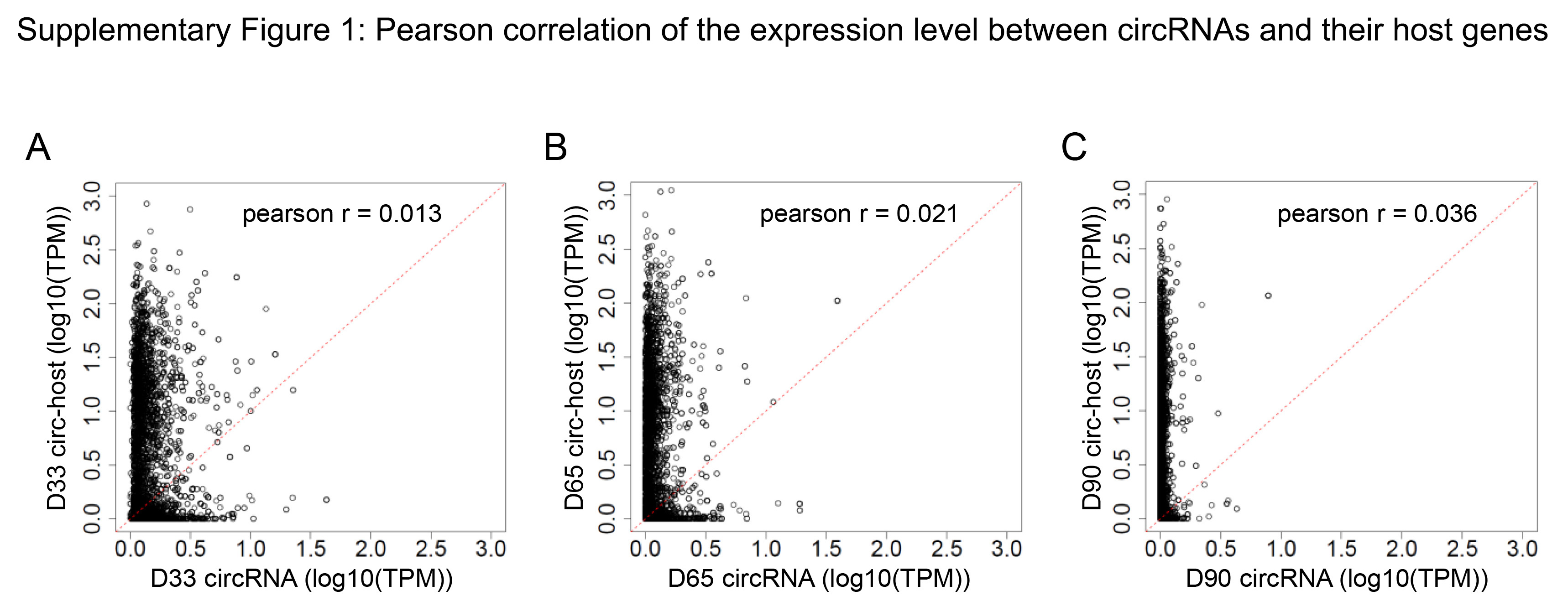

Supplement: Supplementary file 9 [file Image_1.jpg]

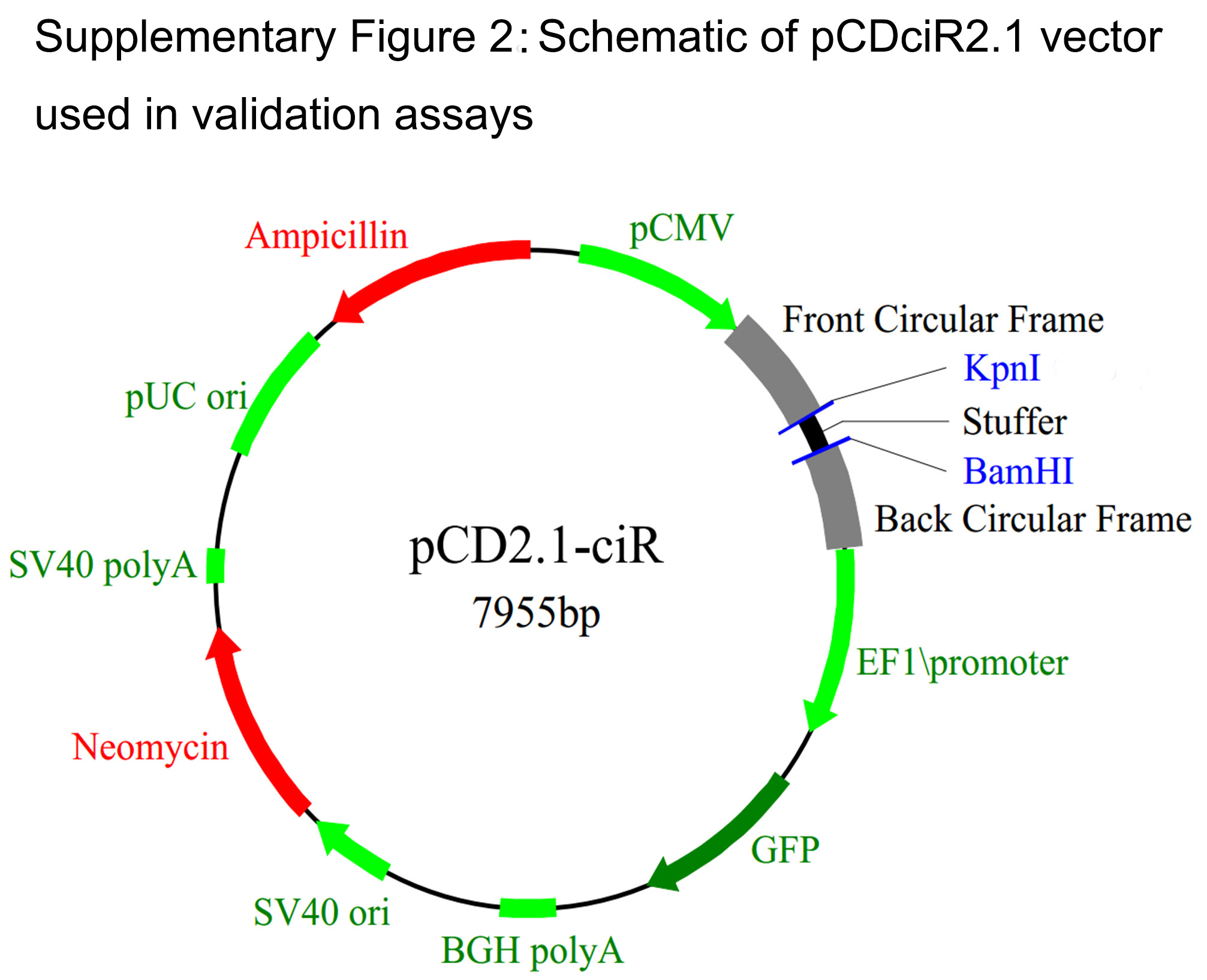

Supplement: Supplementary file 10 [file Image_2.jpg]

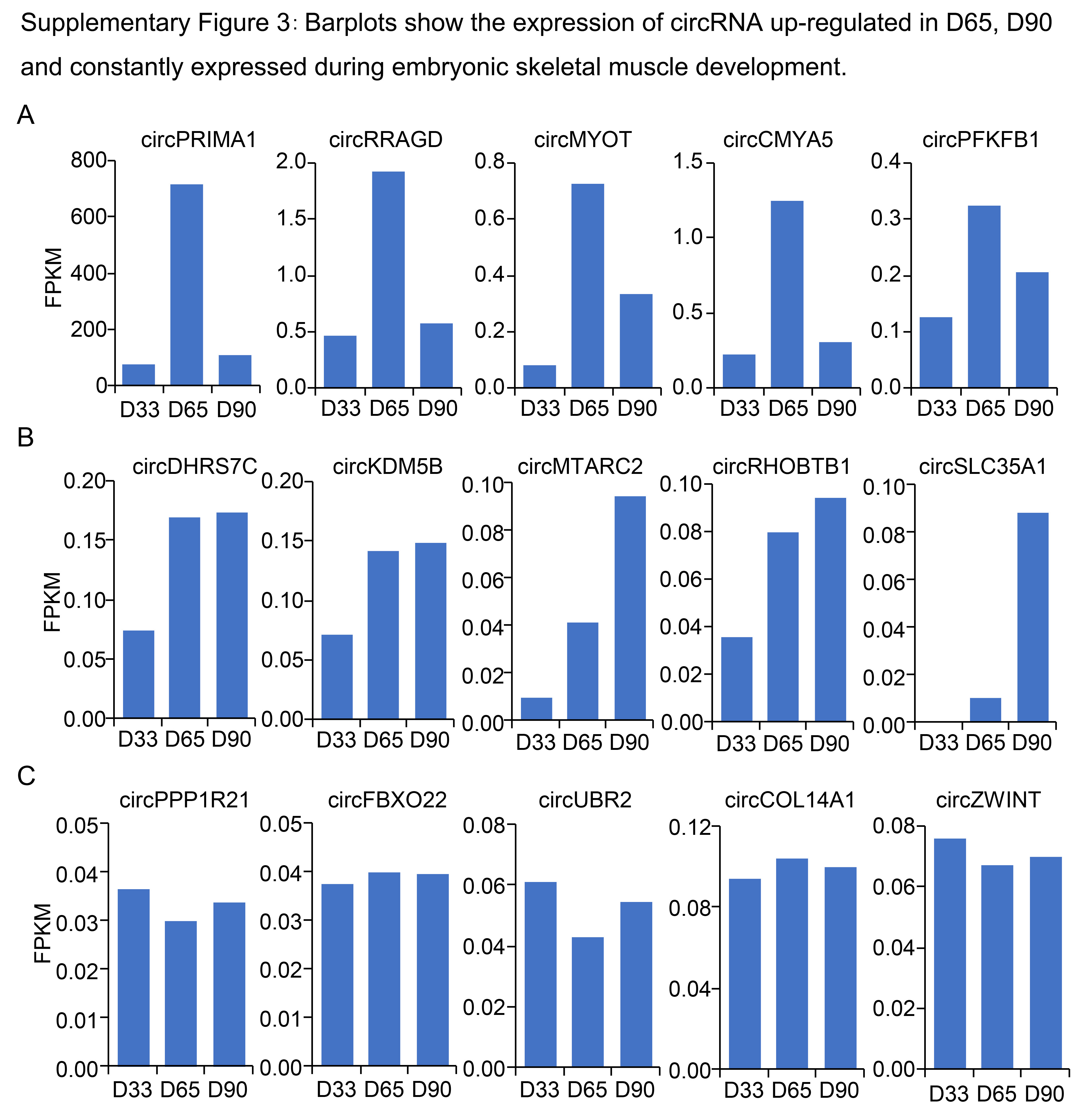

Supplement: Supplementary file 11 [file Image_3.jpg]
